# Case Report: Hypertrophic cardiomyopathy with recurrent episodes of ventricular fibrillation and concurrent sinus arrest

**DOI:** 10.3389/fcvm.2023.1240189

**Published:** 2023-11-03

**Authors:** Jassin Hamidi, Joachim Winter, Rene Weber, Sven Dittmann, Eric Schulze-Bahr

**Affiliations:** ^1^ Institute for Genetics of Heart Diseases, University Hospital Münster, Münster, Germany; ^2^ Department of Cardiology and Rhythmology, Augusta Hospital, Düsseldorf, Germany

**Keywords:** hypertrophic cardiomyopathy, SAN arrest, *MYH7*, ventricular fibrillation, case report

## Abstract

**Background:**

Hypertrophic cardiomyopathy (HCM) is a serious hereditary cardiomyopathy. It is characterized morphologically by an increased left ventricular wall thickness and mass and functionally by enhanced global chamber function and myocellular contractility, diastolic dysfunction, and myocardial fibrosis development. Typically, patients with HCM experience atrial fibrillation (AF), syncope, and ventricular fibrillation (VF), causing severe symptoms and cardiac arrest. In contrast, sinoatrial node (SAN) arrest in the setting of HCM is uncommon. In particular, during VF, it has not been described so far.

**Case summary:**

In this study, we report an 18-year-old woman patient with sudden cardiac arrest due to VF and successful cardiopulmonary resuscitation as the first clinical manifestation of non-obstructive HCM. Subsequently, a subcutaneous implantable cardioverter-defibrillator (ICD) was implanted for secondary VF prophylaxis. However, additional episodes of VF occurred. During these, device interrogation revealed a combined occurrence of VF, bradycardia, and SAN arrest, requiring a device exchange into a dual-chamber ICD. A heterozygous, pathogenic variant of the *MYH7* gene (c.2155C>T; p.Arg719Trp) was identified as causative for HCM.

**Discussion:**

First published in 1994, the particular *MYH7* variant (p.Arg719Trp) was described in HCM patients with a high incidence of premature cardiac death and a reduced life expectancy. Electrophysiological studies on HCM patients are mainly performed to treat AF and ventricular tachycardia. Further extraordinary arrhythmic phenotypes were reported only in a few HCM patients. Taken together, the present case with documented co-existing VF and SAN arrest is rare and also emphasizes addressing the presence of SAN arrest (in particular, during VF episodes) in HCM patients when a distinct ICD device is considered for implantation.

## Introduction

Hypertrophic cardiomyopathy (HCM) is a genetically determined cardiomyopathy characterized by the presence of increased left ventricular (LV) mass and wall thickness. Increasing the stiffness of the LV wall adversely affects the blood flow through the heart chamber by attenuating the tissue elasticity. Phenotypically, severe cardiac arrhythmia, heart failure, or sudden cardiac death may occur, reflecting the development of myocellular disarray and myocardial fibrosis (1). Recent data estimated an HCM prevalence of 1:200–500 (2). In familial HCM, 50%–60% of probands have pathogenic variants of genes encoding cardiac sarcomere proteins; however, the mutation frequency is quite lower in sporadic forms. In addition to the *MYBPC3* gene encoding for myosin-binding protein C, another important cardiac sarcomere protein is the myosin heavy chain beta (encoded by the *MYH7* gene). *MYH7* converts chemical energy into mechanical energy by enzymatic anti-tachycardic pacing (ATP) hydrolyses taking place at the myosin head (3).

Sinus node dysfunction (SND) has a prevalence of 1:600, mostly in cardiac patients above 65 years old with heart failure or coronary artery disease (4). SND is mainly age-dependent but rarely inherited or familial; so far, it is primarily treated by permanent pacemaker implantation (5). One of the first indicators of symptomatic SND is chronotropic incompetence and the inability of the heart rate to properly respond to stress and exercise (6). Other symptoms or ECG signs of SND are sinus bradycardia (<50 bpm), sinus arrest, sinoatrial block and tachycardia–bradycardia syndrome, and syncope or cardiac arrest (7).

With regard to the present HCM case, a concurrent occurrence of ventricular fibrillation (VF) and sinoatrial node (SAN) arrest was documented as an unusual ECG pattern. The repeated appearance of VF and survived sudden cardiac arrest (SCA) supports the severity of the HCM and the existence of the accompanying disturbed sinus node function revealed by sinus arrest at every single event. Furthermore, the resting ECG exhibits sinus bradycardia, which is one of the most common symptoms of SND (7).

## Case presentation

**Figure 1:**
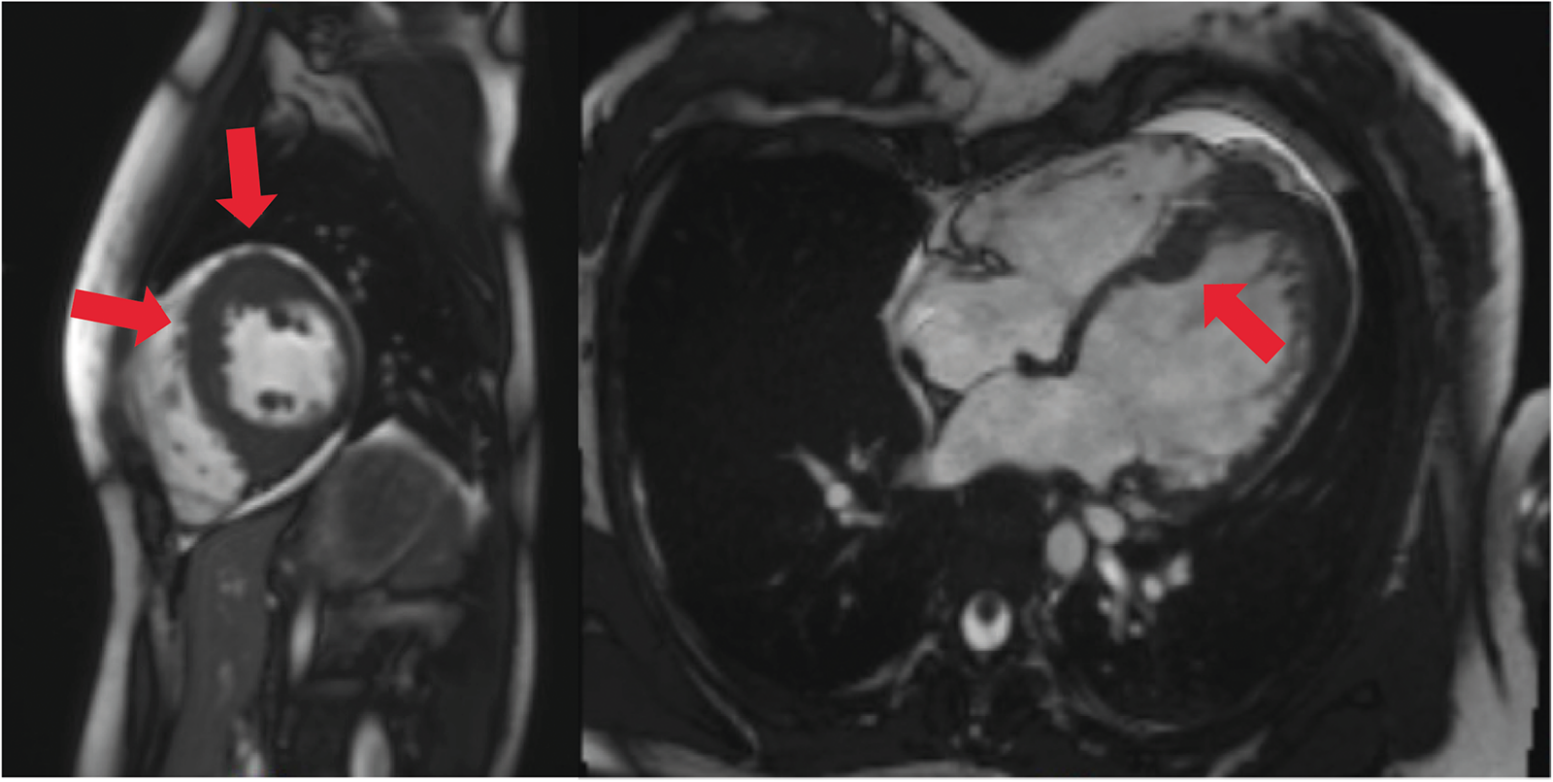
Cardiac MRI, four-chamber view. Depicted long- and short-axis cine images. Red arrows: thickened LV septal and apical wall (up to 20 mm); small apical pericardial effusion.

**Figure 2:**
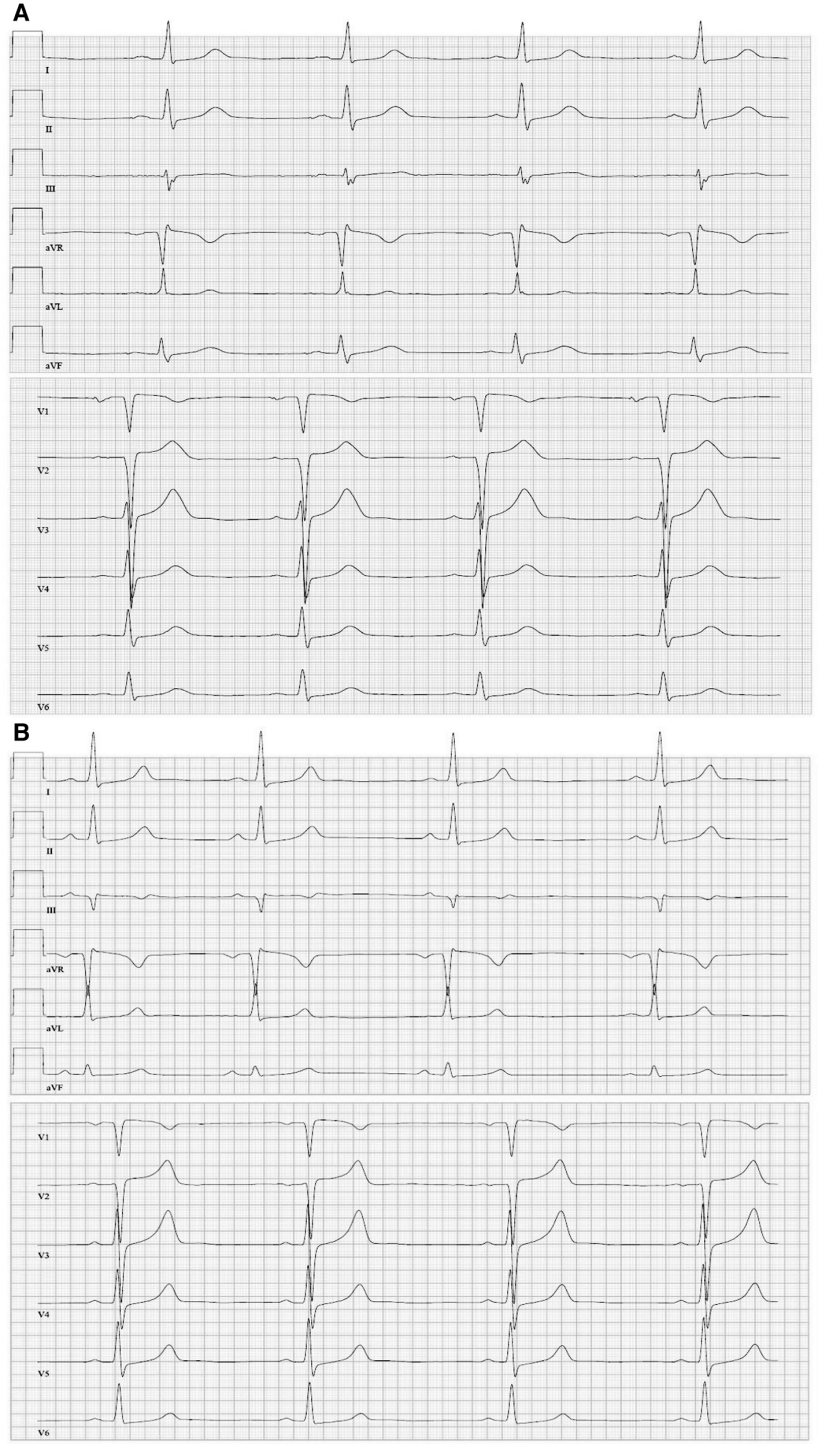
(**A**) Twelve-lead resting ECG (June 2016) (1.25 mg/d bisoprolol) before the first cardiac event depicting sinus bradycardia (45 bpm), regular conduction and repolarization, and tall *T*-waves. (**B**) Twelve-lead resting ECG (January 2018) (medication: 200 mg twice/day amiodarone, 60 mg twice/day diltiazem) with sinus rhythm and a heart rate of 50 bpm.

An 18-year-old woman survived an exercise-related SCA due to VF and immediate cardiopulmonary resuscitation. Initially, an underlying myocarditis was suspected. No indication of heart insufficiency had been detected. The patient has negated other symptoms such as angina pectoris, dyspnea, or previous arrhythmic events, like palpitations or syncopes, before the first hospitalization (Table 1). However, cardiac magnetic resonance imaging was not diagnostic but showed pathologic LV thickening of the anteroseptal and mid-anteroseptal and apical parts (Figure 1, up to 21 mm), leading to the diagnosis of non-obstructive HCM. MRI functional parameters were nearly normal [ejection fraction 53.5%, end-diastolic volume (EDV) 79.1 ml/m^2^, end-systolic volume (ESV) 36.8 ml/m^2^, ejection volume 42.4 ml/m^2^, heart-minute volume 4.9 L/min, LV mass 83.3 g/m^2^]. In addition to the diagnosed non-obstructive HCM with pathologic LV thickening of the anterior, middle, and anterior-septal and apical parts until up to 21 mm, an obstruction in the LV outflow tract (LVOT) could not be confirmed. Gadolinium application showed myocardial late enhancement and fibrosis, particularly in the hypertrophied LV sections. Ajmaline testing was negative for Brugada syndrome. Initially, a subcutaneous ICD (S-ICD) was implanted. Striking was a post-shock pacing with 22 pulses of 26 s duration. We interpret this as a depression of the sinus node due to the 65-J shock. A medication consisting of 1.25 mg/day bisoprolol and 1.25 mg/day ramipril was initiated upon discharge. The baseline ECGs showed a normal but bradycardic sinus rhythm (45 bpm; Figure 2A) and conduction at rest without any disturbances of repolarization or LV hypertrophy.

**Table 1:** Basic information.

Basic information	
Sex	Female
Age	18 years
Previous medical history	None
Alcohol intake	No
Asthma	No
Blood pressure (systolic/diastolic)	98/59 mmHg
BMI	24.5
Chronic kidney disease	No
COPD	No
Coronary heart disease	No
Creatinine	0.86 mg/dl
Diabetes	No
Dialysis	No
Drug abuse	None
Family history of stroke	None
Gastrointestinal bleeding history	None
Gout	No
Heart failure	No
Hemoglobin	13.5 g/dl
History of cancer	None
HIV status	Negative
Hypertension	No
Liver cirrhosis	No
liver steatosis	No
Smoker	No
Stroke	No

BMI, body mass index.

A stress-induced syncope event occurred 16 weeks after the first event. Interrogation of the S-ICD revealed a successful termination of VF by a single S-ICD shock, followed by a sinus node arrest of nearly 90 s hereafter. Subsequently, the S-ICD was replaced by a dual-chamber transvenous ICD system with correct positioning and regular function of the device leads. At discharge, the daily medication of the patient included 2.5 mg bisoprolol, 1.25 mg ramipril, and 300 mg magnesium and tromcardin.

**Figure 3:**
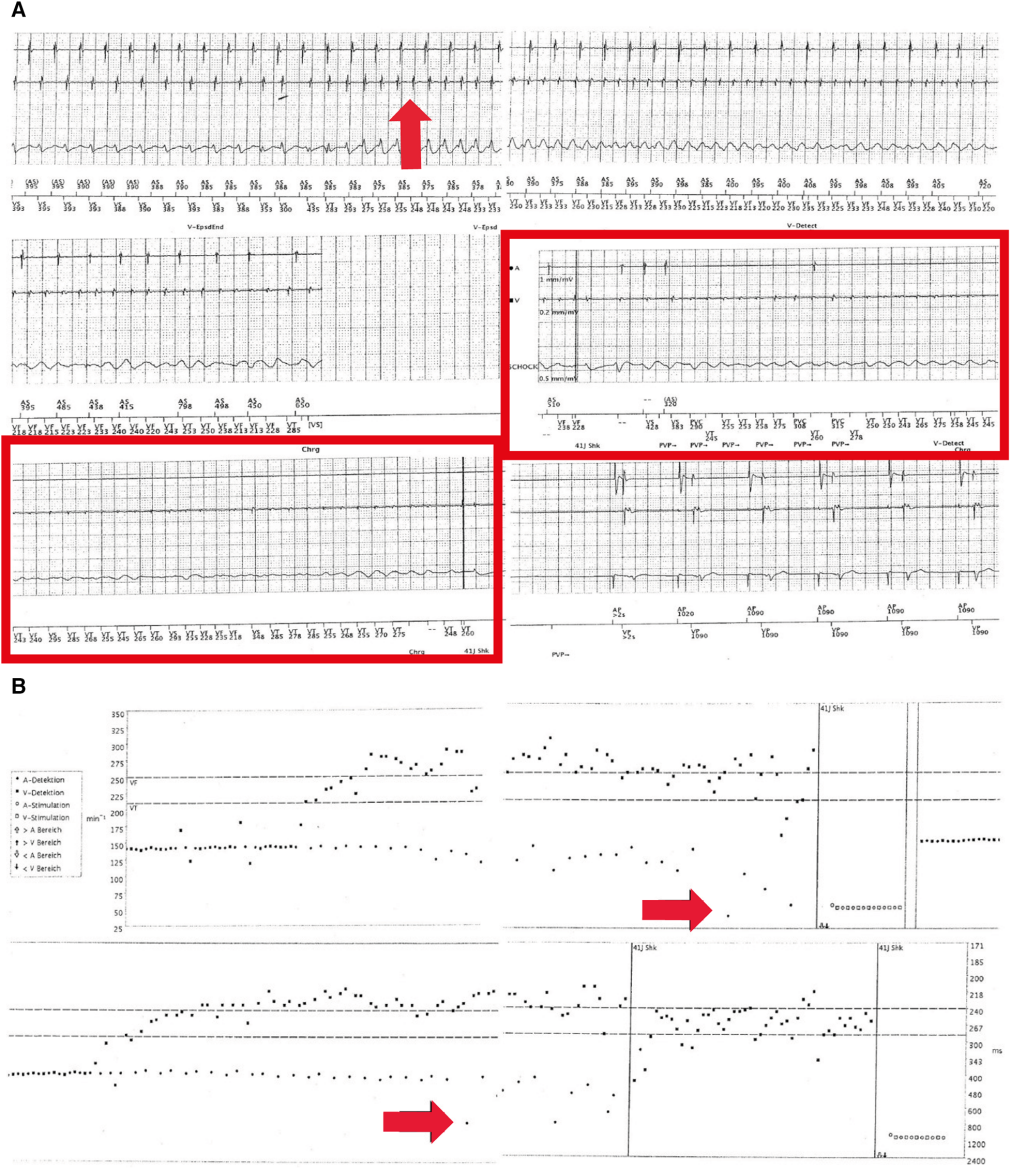
(**A**) Electrograms during the exercise of the dual-chamber ICD in August 2016. Sensing of a spontaneously occurring ventricular tachycardia (approximately 230 ms, 260 bpm, red arrow in the left upper panel) can be observed. Of note, initially regular atrial sensing (approximately 400 ms or 150 bpm; exercise-related) (upper two leads in the left upper panel) but further degeneration into VF (red arrow in the right upper panel) and sinus bradycardia and arrest (middle panels, red rectangle) can be seen. In the lower panel, ventricular shock (41 J) and hereafter atrial pacing (1,000 ms or 60 bpm) and ventricular sensing indicated a recurrence of regular ventricular activity. (**B**) Histograms corresponding to (**A**). Spontaneously occurring ventricular tachycardia and fibrillation (upper panel) arising from sinus tachycardia (exercise, 150 bpm) (red arrow in upper panel) can be observed. In the lower panel, a red arrow indicates the occurrence of sinus bradycardia and sinus node arrest during VF. Vertical lines indicate an ICD shock (41 J) and atrial stimulation.

After another period of 10 weeks, physical exercises again triggered another VF episode, which could be adequately terminated by the dual-chamber ICD. Subsequent device interrogation revealed adequate sensing of VF after a short period of previous ventricular tachycardia. Of note, atrial sensing (AS) showed an initially regular atrial activity aggravating into sinus bradycardia and cardiac arrest due to complete atrial asystole (AA) but still concurrent VF of low amplitude. This illustrates a rare ECG registration and co-event of VF and SAN arrest or AA (Figures 3A,B).

Using a multi-gene-panel sequencing approach, a pathogenic and known heterozygous non-synonymous variant (p.Arg719Trp) (ACMG class 5: PS3, PS4_M, PM1, PM2, PP3) of the *MYH7* gene was identified. This variant was reported in >10 unrelated HCM patients and is nearly absent in controls (gnomAD: MAF 0.003%), further supporting its pathogenicity. Other NGS panel genes for inherited arrhythmia forms or cardiomyopathies showed no further pathogenic variant.

**Figure 4:**
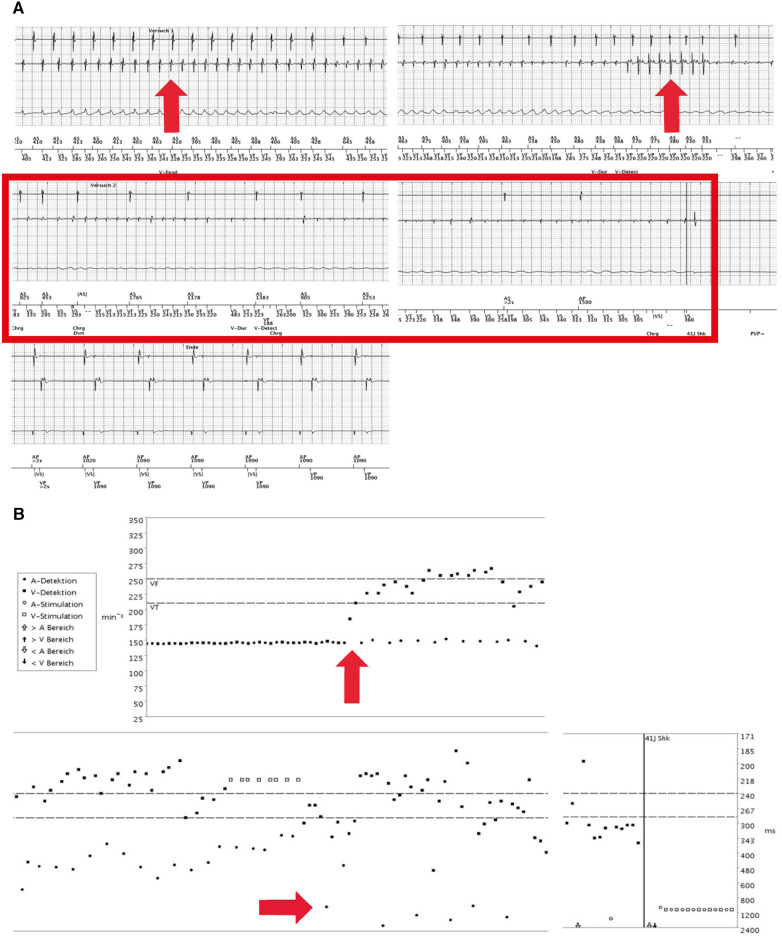
(**A**) First electrograms of the dual-chamber implantable cardioverter-defibrillator (ICD) (during physical exercise, September 2017). Re-occurrence of a VF episode (red arrow in left upper panel), again induced by physical exercise. Remarkably, during persisting VT, the atrial sensing aggravated again into sinus bradycardia and finally sinus node arrest until the second ICD shock, hereafter, atrioventricular pacing (right middle and left lower panels). (**B**) Electrograms (September 2017). ICD sensing shows a VF at the ventricular level and atrial tachycardia that further decelerates (red arrows). Vertical lines indicate ICD shock (41 J each) to restore regular and paced rhythm and rhythm restoration but re-occurrence of VF and sinus node arrest.

A beta-blocker therapy was established to prevent exercise- or stress-induced fatal arrhythmic events. Nevertheless, another exercise-induced VF episode occurred when the patient was climbing stairs, 83 weeks after the first event. Device data initially demonstrated sinus tachycardia with cycle lengths from 385 to 400 ms in addition to ventricular extrasystoles (VES). Notably, interrogated ICD data again demonstrated a co-occurrence of ventricular tachycardia degenerating into VF and accompanying a decelerating AS to sinus bradycardia followed by asystole (Figures 4A,B). Although this episode of VF was regularly terminated through the first ICD shock, VF re-occurred in the following minutes and was again accompanied by sinus bradycardia and finally sinus asystole. Another two ICD shocks, including ineffective defibrillation, were necessary to adequately terminate the VF/AA. However, fast shortening of the ventricular cycle length and severe degeneration of the signal amplitude in the EGM channel with partial undersensing of single events exposed an ineffective detection sensitivity. The dual-chamber ICD was reprogrammed to reduce the electrode threshold in the right ventricle to 0.3 mV because the minimum amplitude of the last VF episode was 1.4 mV.

Another VF episode occurred during a psychotherapeutic appointment 3 months later. Notably, it initially emerged during the resting phase without physical exercise, followed by inadequate termination via the ICD through several shocks. Altogether, three particular episodes of VF were registered in the ICD data. The first episode was defibrillated twice, the second one was successfully terminated through ATP, and the third was inadequately terminated by another single ICD shock. During the approximately 7 min of lasting arrhythmia episode, cardiopulmonary resuscitation was conducted until emergency physicians arrived. After intravenous administration of amiodarone (300 mg), the ICD effectively terminated the VF episode by a shock intervention, and a reprogramming of the dual-chamber ICD was conducted due to undersensing.

During hospitalization, amiodarone treatment was initiated (initial saturation treatment and then 200 mg/day). Furthermore, diltiazem therapy (2 × 60 mg/day) was initiated. No further severe cardiac arrhythmic events have been detected since then (Table 2).

**Table 2:** Timeline.

Timeline	
Week 1	SCA due to VF as the first clinical symptom. After successful cardiopulmonary resuscitation, the diagnosis of HCM was made
Week 4	S-ICD implantation for secondary SCA prophylaxis
Week 16	Recurrence of a VF episode during stress, sufficiently terminated by S-ICD; subsequently, documented sinus node arrest and asystole for >90 s
Week 17	Device exchange of the S-ICD to a dual-chamber (DDD-) ICD
Week 27	Genetic testing revealed a pathogenic heterozygous non-synonymous variant (p.Arg719Trp) of the *MYH7* gene
Week 27 + 1 day	Re-occurrence of stress-induced VF episode (without beta-blocker therapy) and adequate shock termination by the ICD device (Figures 3A,B); documented co-existing SAN arrest during VF; initiation of beta-blocker treatment
Week 83	Exercise-induced VF during beta-blocker therapy and need for three ICD interventions to restore normal rhythm; partially ineffective defibrillation with rapid shortening of the ventricular cycle length and severe degeneration and low-amplitude signaling in the EGM; subsequently, the ICD was reprogrammed to increase sensitivity and reduce the detection threshold in the right ventricle (to 0.3 mV; minimum VF amplitude: 1.4 mV) (Figures 4A,B)
Week 95	An initial emerging VF storm event occurred during a psychotherapeutic consultation, followed by insufficient termination via the ICD; cardiopulmonary resuscitation and intravenous administration of amiodarone, together with external defibrillation to terminate VF; initiation of oral amiodarone and diltiazem
Follow-up	No VF recurrences have been detected over 4 years

Notably, since April 2022, the patient has suffered from the first symptoms of hyperthyroidism [thyroid-stimulating hormone (TSH) < 0.01 µIU/ml, anti-Tg-RO = 1,771.0 IU/ml, anti-TPO Roche = 312.0 IU/ml]. Although amiodarone-induced functional abnormalities in thyroid homeostasis are quite common, an autoimmune-induced hyperthyroidism could not yet be excluded. Nevertheless, thyreostatic therapy was initiated with thiamazole and Irenat. Considering the young age of the patient and the long-term toxicity of amiodarone, a transition to another less toxic antiarrhythmic medication such as sotalol would be preferable, especially to reduce the risk of hyperthyroidism. A medication change has not been conducted yet due to severe psychological concerns of the patient during the follow-up related to a replacement of amiodarone

The family history for HCM, SCD, or SCA was unremarkable; a cascade of clinical and genetic testing was offered to the family but was not completed.

## Discussion

First described in 1994, the *MYH7* p.(Arg719Trp) mutation was initially associated with a high incidence of premature death and, thereby, a significant decrease in average life expectancy (8). *In silico* prediction models, particularly designed for the assessment of non-synonymous variants in HCM, confirmed the relevance of the variant. Furthermore, this underlines previous findings (9) because a significant increase in disease-associated missense variants could be localized to the spherical region where the p.(Arg719Trp) variant is located (10).

Remarkably, around 31% of patients with *MYH7* mutations have ventricular conduction system impairments (mainly bundle branch blockade). However, cases with documented concurrent decelerating AS and asystole in combination with VF are rare in the literature. This phenotypic discrepancy between atrial and ventricular rhythm represents some uniqueness in the present case and emphasizes considering SAN arrest in the setting of HCM and VF before deciding to implant a distinct ICD device. In this line, invasive electrophysiological studies of 155 HCM patients revealed an abnormal sinus node recovery time (>1,500 ms) in 7% (11). Moreover, the co-existence of HCM and SND as primary heritable diseases is rarely described; in a single, larger study, SND (including sinus bradycardia and sinus node arrest) was described in 17% of an HCM cohort of 101 enrolled patients (12).

Deleterious *MYH7* missense variants and other overlapping phenotypes, e.g., HCM with LV non-compaction (LVNC), have been reported (13). Thus, considering the heterogeneous arrhythmic cardiac phenotype in this case, it remains unresolved whether the pathogenic *MYH7* missense variant can directly result in such an extraordinary arrhythmia presentation as seen here. Of note, longer episodes of VF may lead to a high secretion of adenosine that may compromise regular SAN function. The latter may be in line with the clinical observation that aminophylline may be useful in restoring cardiac rhythm in this setting (14–16).

Pathogenic MYH7 variants, as described in the present case, belong to the most common genetic etiologies for HCM, with an involvement of about 15%–25% in all HCM patients. Clinical manifestations can also vary depending on the specific gene and causative variant. They can reach from ventricular tachycardia (VT) or supraventricular tachycardia and syncopal episodes to minor severe symptoms such as chest pain and dyspnea on exertion (17). Recently, Higuchi et al. (18) reported an association between HCM and first-degree atrioventricular block (AVB) as another symptomatic manifestation by analyzing 414 patients with HCM. Among these, 96 patients were diagnosed with AVB, representing 23% of all enrolled patients.

Taking into account the implanted dual-chamber (DDD-) ICD device, one could suggest an Atrium, Atrium, Inhibited (AAI) pacing mode for appropriate SND treatment (19). Nevertheless, this approach might not be applicable to address the SAN arrest in the described case because the episodes of SAN arrest only occur simultaneously with VT and VF, which would be inadequately treated by single atrial pacing. Further, AAI pacing was shown to be the opportune mode for patients suffering from SND and coronary heart disease, atrial fibrillation (AF), or atrioventricular (AV) block but not for HCM patients. Overall, AAI pacing was assumed to have a higher suppressive effect on atrial arrhythmias than ventricular pacing (20).

SND in the setting of VF episodes and, in particular, hereafter manifesting as SAN arrest may lead to the necessity of a dual-chamber transvenous ICD system to treat atrial and ventricular arrhythmias. The clinical severity—in contrast to the moderate LV hypertrophy—due to the extraordinary arrhythmic phenotype also required additional medical, antiarrhythmic treatment.

Remarkably, the identified pathogenic *MYH7* variant (p.Arg719Trp) has already been reported to cause unexpected arrhythmic events, e.g., in the case of a young 12-year-old girl. The patient suffered from dizziness and chest discomfort while walking around. Furthermore, she experienced syncopal attacks with a heart rate drop to 40 bpm. ECG exhibited an AVB of the third degree (AVB III°) in combination with a complete left bundle branch block. Whole exome sequencing also identified the heterozygous missense variant of *MYH7* (p.Arg719Trp) and no other variants of arrhythmia genes (21). Considering this described phenotype is related to the particular *MYH7* variant, atrial pacing might be suitable for an advanced therapeutic approach. So far, the “coupled clock” is a widely accepted model of SAN automaticity and proposes a functional interplay (coupling) between the activity of pacemaking ion channels of the cell plasma membrane and the activation of the Na^+^/Ca^2+^ exchanger (NCX1) by spontaneous diastolic Ca^2+^ release from the sarcoplasmic reticulum (SR), mediated by RYR2 receptors. Hsieh et al. (22) established two human-induced pluripotent stem cell (hiPSC) lines carrying an MYH7 variant (p.Arg723Cys); at mature stage, the generated derived mutant cardiomyocytes displayed HCM-consistent phenotypic characteristics, such as hypertrophy, altered calcium handling and metabolism, and also arrhythmias. Notably, hiPSC-derived cardio­myocytes investigated before emerging of known HCM characteristics exhibited dysregulated extracellular matrix (ECM) remodeling, limited formation of focal adhesion expressed by interrupted cell–ECM adhesion, and altered integrin expression. Altered intracellular calcium handling due to *MYH7* variants may also influence the Ca^2+^-dependent activity of proteins involved in SAN automaticity.

### Study limitations

SAN functional parameters, in particular SNRT and SACT, HR parameters (max/min) during day and night, and HRV (e.g., before and after VF storm and successful treatment) were not available. Thus, it could not be determined whether SAN dysfunction alone led to any symptoms. Furthermore, the evaluation of SAN function upon adenosine bolus and subsequent aminophylline or theophylline treatment and also atropine treatment to restore SAN rhythm could not be analyzed because the focus was set on anti-tachycardic intervention (15, 16). Consequently, due to the lack of specific SAN function data, it must be acknowledged that SAN arrest (SAN pacemaker/automaticity arrest) from the SAN exit block and conduction impairments cannot be distinguished in this case.

### Future directions

The heterogeneous and unexpected phenotype in this case represented by episodes of VF and concurrent SAN arrest may lead to the necessity of a dual-chamber transvenous ICD system to treat atrial and ventricular arrhythmias simultaneously. Thus, when approaching patients with genetically determined cardiomyopathies such as HCM, in particular, with causative *MYH7* variants, a treatment for bradycardic arrhythmias should also be considered before deciding to implant a distinct ICD device.

## Conclusion

Although the underlying *MYH7* mutation for HCM and (recurrent) VF has been known for almost 30 years, the particular phenotype with SAN arrest has not been noted in detail. Assessing not only for ventricular but also bradycardic arrhythmias might be useful in particular HCM patients and distinct gene mutations.

## Data Availability

The original contributions presented in the study are publicly available. This data can be found here: https://www.ebi.ac.uk/ena. Study name: 877a8bc8-06ec-4f7a-a195-3b7ed94e864f. Accession numbers: PRJEB64497; ERP149670.The deposited data will become public on the 15 November, 2023.
